# Cultural adaptation of the Savvy Caregiver program with the Native Hawaiian community

**DOI:** 10.1002/alz.088252

**Published:** 2025-01-09

**Authors:** Alexandra Jackson, Adrienne Dillard, B. Puni Kekauoha, Mahealani Austin, Errol Kia'i Lee, Poki'i Balaz, Natlie Dutro, Scott Okamoto, Kenneth Hepburn, Jordan P. Lewis, Kelly O'Sullivan, Paige Mayeda, Angeliyah Kahoku Dutro, Raven Weaver

**Affiliations:** ^1^ Pacific University, Forest Grove, OR USA; ^2^ Kula no na Po'e Hawaii, Honolulu, HI USA; ^3^ Papa Ola Lokahi, Honolulu, HI USA; ^4^ Obama Foundation, Columbia University, NY USA; ^5^ Ka ʻAha Lāhui O ʻOlekona Hawaiian Civic Club, Portland, OR USA; ^6^ University of Hawai'i, Honolulu, HI USA; ^7^ Emory University Nell Hodgson Woodruff School of Nursing, Atlanta, GA USA; ^8^ Memory Keepers Medical Discovery Team, University of Minnesota Medical School, Duluth campus, Duluth, MN USA; ^9^ Washington State University, Pullman, WA USA

## Abstract

**Background:**

To promote caregiver health and reduce burden, the Savvy Caregiver Program (SCP), an evidence‐based caregiving intervention, was adapted with a Native Hawaiian (NH) community in Hawaiʻi. The adaptation process occurred prior to pilot testing in two phases: 1) the preliminary adaptation by a community action board (CAB) and mentorship team and 2) pre‐pilot testing and expert validation with NH adults. The preliminary adaptation, titled ʻAuamo Kuleana O Nā Maʻi Poina (ʻAuamo Kuleana), aimed to include Hawaiian values, language, proverbs, and culturally relevant examples while maintaining the core components of the program. The CAB also created new videos with cultural foods, mirroring the existing training videos. To continue refining the adapted program prior to pilot testing, we pre‐piloted the curriculum and invited participants to describe their experiences and provide feedback.

**Method:**

Individuals who participated in the 7‐week pre‐pilot of ʻAuamo Kuleana (n = 6) were asked to complete a focus group or interview to share their experiences and provide feedback. The CAB was invited to analyze the qualitative data using thematic analysis.

**Result:**

Five pre‐pilot participants attended a focus group or interview. Only one participant was actively providing care, the other participants were interested in proactive training to support their community. This was a common theme: caring as a community. Participants shared positive feedback about the program; most participants attended all sessions and read the caregiver manual. All participants preferred to meet in‐person and appreciated the revised curriculum. Two suggestions emerged: include all family members in the program to be in alignment as a ʻohana (family) in providing care; and add information about planning for their own care.

**Conclusion:**

There were high rates of satisfaction and engagement and the pilot test of ʻAuamo Kuleana proceeded without additional changes. The suggested changes from the pre‐pilot required modifications to program core components and were not incorporated at this stage. However, it may be important to consider how to incorporate cultural aspects of caring, like participants' interest in proactively learning about dementia and caregiving, caring as a community, and family‐centered caregiving (rather than dyadic caregiving), in future caregiving interventions co‐created with the NH community.

